# A qualitative exploration of early assessment of innovative medical technologies

**DOI:** 10.1186/s12913-018-3647-z

**Published:** 2018-11-06

**Authors:** Iben Fasterholdt, Anne Lee, Kristian Kidholm, Knud Bonnet Yderstræde, Kjeld Møller Pedersen

**Affiliations:** 1 CIMT - Centre for Innovative Medical Technology, Odense University Hospital, Sdr. Boulevard 29, Entrance 102, 3rd floor, 5000 Odense C, Denmark; 2 Department of Medical Endocrinology, Odense University Hospital, Odense, Denmark; 3 Department of Business and Economics, University of Southern Denmark, Odense, Denmark

**Keywords:** Early assessment, Health technology assessment, Interview, Innovation

## Abstract

**Background:**

Hospitals increasingly make decisions about early development of and investment in innovative medical technologies (IMTs), but at present often without an early assessment of their potential to ensure selection of the most promising candidates for further development. This paper explores how early assessment is carried out in different health organisations and then discusses relevant learning points for hospitals.

**Methods:**

A qualitative study design with a structured interview guide covering four themes was used. Content analyses of interview notes were performed covering four predetermined themes: context, basis for decision-making, process and structure, and perceptions. A fifth theme, handling cognitive bias, was identified during data analysis.

**Results:**

A total of 11 organisations participated; eight from the private health industry and three public hospitals. The interviews identified four areas in which early assessment is performed in similar manner across the studied organisations and four areas where differences exist between public hospitals and private organisations. Public hospitals indicate a lower degree of formalised early assessment and less satisfaction with how early assessment is performed, compared to private organisations. Based on the above findings, two learning points may carry promise for hospitals. First, having dedicated prioritising committees for IMTs making stop/go decisions. This committee is separate from the IMT development processes and involved staff. Secondly, the committee should base decisions on a transparent early assessment decision-support tool, which include a broad set of domains, is iterative, describes uncertainty, and minimise cognitive biases.

**Conclusions:**

Similarities and differences in the way early assessment is done in different health organisations were identified. These findings suggest promising learning points for the development of an early assessment model for hospitals.

**Electronic supplementary material:**

The online version of this article (10.1186/s12913-018-3647-z) contains supplementary material, which is available to authorized users.

## Background

As part of the increasing technology development and digitalisation of the healthcare systems, hospitals establish centers for innovation [1, 2] and engage in designing, developing, and testing innovative medical technologies (IMTs). IMT in general include medical devices, medical/surgical procedures, processes of care, and clinical health information systems, e.g. an app for discharge early postnatally [3], an automatized medical ultrasound examination and interpretation robot [4], telemedicine training after hospitalisation with severe chronic obstructive pulmonary disease [5], and 3D camera for ulcer treatment and care [6].

Traditionally, the process of development of IMTs/pharmaceuticals takes place in a pharmaceutical, medical device or IT industry setting. After industry product development and testing hospitals make a purchasing decision, i.e. decide to start large-scale clinical tests or adopt the IMT. The agreement between the two stakeholders, industry and hospital, hence was: “hospitals buying” and “industry developing and delivering” [7]. However, we argue that this division of labor is gradually changing because of a national and international focus on promoting public-private partnerships [8], and the aforementioned creation of dedicated innovation units in large hospitals. Consequently, hospitals join the process earlier and are increasingly involved in developing IMTs, either internally or by working in close collaboration with industry.

IMTs often require interdepartmental cooperation, considerable costs and large investments, and significant modification in features, design, or properties before widespread clinical use or adoption. Therefore, early assessment of early stage IMTs is needed and may provide hospitals with the following three benefits: 1) The ability to discriminate potentially promising IMTs from less advantageous ones early in the process, and thus avoiding misallocating public resources [9], 2) Early influence on an IMT’s value proposition [10], and 3) A system that safeguards against “pro-innovation bias” [11], i.e. the perception that any innovation (IMT) will lead to increased performance, often due to unrealistic assumptions and optimism bias [12].

**Fig. 1:**
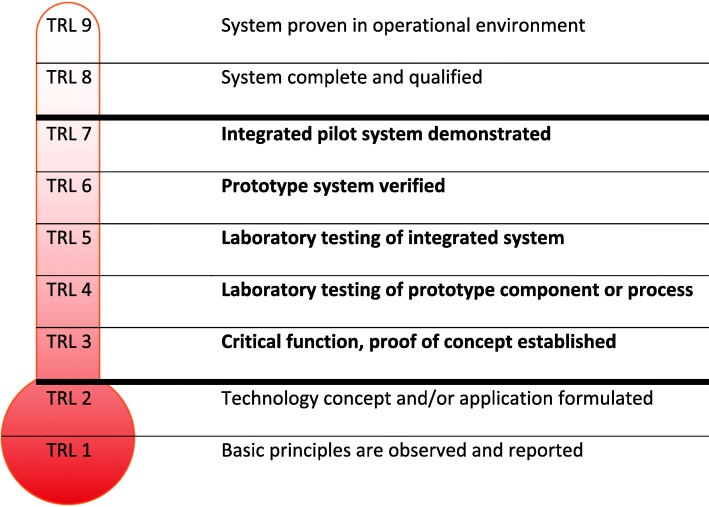
Defining early with Technology Readiness Levels (TRL) based on early NASA model

If formal evaluation of IMT is performed, it is often conducted late in the developmental process [13, 14] using traditional approaches like Health Technology Assessments (HTA), hospital-based HTAs, cost-effectiveness analyses, or clinical trials. Using a popular scale for assessing the maturity level of a particular technology this corresponds to level 7 to 9 in Fig. 1 (where 1 is the lowest maturity and 9 is the highest). In contrast, early assessment of IMTs normally takes place between level 3 to 7 in terms of technological maturity [15], Fig. 1.

However, assessment of investments in IMTs early in the development process is different from the task of assessing mature technologies at later stages deciding whether adoption is warranted. Data from early phases will by nature be limited and a high level of uncertainty concerning clinical, patient, economic, and organisational effect exists. Thus, early assessment is based on feasibility, pilot, demonstration, or initial effect data generated for the IMT and exclude large-scale testing or traditional clinical research.

Currently, the best approach for early assessment of the potential of IMTs in hospitals is unknown. Thus, there is a need to develop a model for early assessment in hospitals. This study provides the first, qualitative step in this endeavour by exploring how early assessment of IMTs is performed and perceived in different health organisations and subsequently discuss relevant learning points for hospitals.

## Methods

A qualitative exploratory study was used to investigate how early assessment of expected potential/value of innovation projects is performed in three sectors: private pharmaceutical and medical device industry and in public hospitals. By choosing an exploratory research design we basically explore the research question, leaving room for further conclusive research aimed at providing (more) final findings [16]. Data collection took place from August 2014 – June 2015. An interview guide was developed and eleven interviews were conducted: ten face to face and one as a telephone interview, lasting one to two hours.

A mix of convenience and the purposive sampling procedure of maximum variation [17] was used to select companies and hospitals. Participants for interviews were identified through the authors’ networks according to three pre-specified selection criteria: probable experience with early assessment, sector, and size of organisation. Thus, the study population consisted of experts and professionals from large device and pharmaceutical companies and university hospitals, working in an R&D or innovation unit having experience with early assessments of IMTs. Apart from one case, the interviewer (IF) had no prior relationship with the participants. Recruiting new organisations was an iterative process and continued until the themes seemed exhausted in the way that new information was minor [18].

The developmental process in pharmaceutical and medical devices is very competitive and highly sensitive to business interests. In the literature it is mentioned that internal strategic decisions, i.e. early assessments, are rarely published and take place behind closed company doors [19, 20]. Hence, due to confidentiality concerns it was decided that the interviews were not recorded knowing that from a methodological point of view it would be a weakness but considered necessary to gain access to key persons. Instead, detailed notes were taken during the interviews. At the start of each interview, participants were informed about the objective of the study and that their answers were anonymous.

### Development of the interview guide

As advocated by Miles, Huberman and Saldana [21] initially a rudimentary conceptual framework was built to orient the study [see Additional file 1], and key factors relevant to early assessment were described resulting in the four research themes: context, basis for decision making, process and structure, and perceptions.

This process included a preparatory literature search later published as a review by our group [22]. From this work, a toolkit for the identification and assessment of new and emerging health technologies [23] motivated questions on frequency of updates of IMT, time horizon, templates, and questions on satisfaction with the process. Further, a planned study of early economic evaluation of emerging health technologies [24] inspired the questions on the basis for decision making, including the importance of risk/uncertainty. Also, valuable inputs for the interview guide were solicited through discussions with a diverse and skilled supervisor team including two health economists and one medical doctor.

Based on this an interview guide was developed and then subsequently piloted by a health technology assessment consultant outside the author team. The questions were generally well understood and reductions and some minor changes to reflect more open questions were included. Table 1 shows the four predetermined research themes and a rough sketch of related questions. The complete interview guide and details about its development are published elsewhere [25].

**Table 1:** Overview of research themes and questions regarding early assessment

Theme	Questions
1) Context	What is the time horizon in the developmental process? How many technologies/ideas do you evaluate in one year – rough estimate of the size of your portfolio of projects?
2) Foundation/basis for decision making	Is there an approach/template for early assessments? Do you use a fixed/systematic procedure? What are the dimensions/inputs in the early assessment - Which components are used? • Do you use qualitative data/information? • Do you use quantitative data/information? • Is there an economic calculation (even simple) in the pilot phase? • Are expected clinical effects included early – for example mortality, amputations, admissions? Do you perform risk/uncertainty assessments?
3) Process and structure of early assessment	Which phases are used in the developmental process? Are there stop/go decisions in the process of development – what are they based on? Do you update your assessments regularly – how often? Who decides whether to continue an innovation - who makes the go/no-go decision? How do you decide which effects to include? Success rate^a^: Which proportion of projects/ideas survives the “pilot phase” and achieves routine use or commercialisation?
4) Perceptions	Relevance and satisfaction with the used method? What are the pros? And what are the cons (wish list)?

^a^Definition of success rate: The proportion of an organisation’s R&D product portfolio that “survives” the early phases in the developmental process and becomes a “success”. While success in the public sector is defined by the product achieving routine use, in the private sector the product must be commercialised or launched on the market to be termed a success

### Data analysis

Qualitative content analysis was used to investigate both the obvious content and underlying themes and meanings of the phenomena studied [21]. Conceptual (deductive) coding in the form of codes from the four predetermined themes was used and to a minor degree supplemented by (inductive) data-driven coding identifying a new theme [21, 26]. The following components were present in the data analysis: data reduction, data display and interpretation/conclusion drawing and validation, forming a cyclical process moving between the components [21]. Through this process, an extended text was reduced and ordered to allow careful comparisons, detection of differences and noting of patterns and themes [21, 26], as described in the following three stages.

**Fig. 2:**
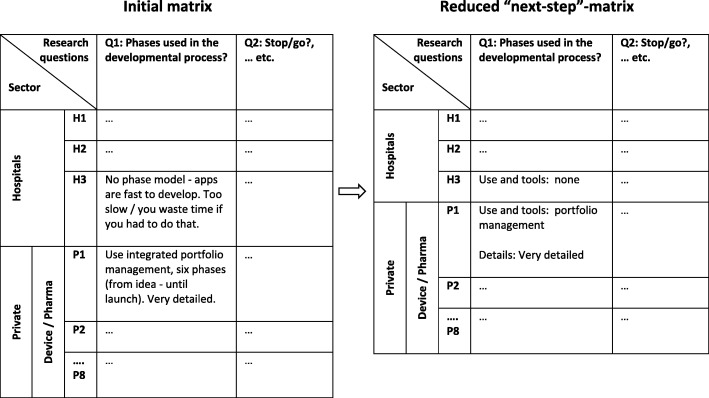
Data display and data and meaning condensation for theme 3, question 1. Note: Left part: Raw data, right part: Reduced data

At stage 1 an initial data reduction took place, capturing the essence of the interview based on notes taken during interviews. Each interview was then assigned a name in arbitrary order from H1-H3 for public hospitals and PI-P8 for private organisations. In the second stage a descriptive matrix was designed to display data [21] and this essential construct is visualized in the left part of Fig. 2.

A sector and variable-ordered matrix was constructed for each of the four themes and with the organisations (H1 to H3 and P1 to P8) as rows and research questions as the columns. Hence, the initial matrix was built on the themes and research questions chosen by the authors (Table 1). Next, reading through the interview data, one interview at a time, IF placed individual pieces of data in the corresponding matrix-cell until all data were aggregated according to the four themes and categorised according to the sub-questions. During this process and as some data did not correspond to the predefined themes a new theme labelled cognitive bias and a fifth matrix was constructed. Cognitive biases are described as ‘systematic pattern of deviation from norm or rationality in judgment, whereby inferences about other people and situations may be drawn in an illogical fashion’ [12].

In stage 3 the content of the theme matrices were investigated. Each matrix was read vertically first, i.e. column-wise (for specific questions), giving a picture of the answers to each question across different organisations. Next, horizontal reading gave a picture of each organisation’s answers across all questions in a theme. This method provided useful means for assessing patterns, i.e. similarities and differences between organisations, between the three sectors (and eventual between public and private). At this stage a further data/meaning condensation took place and a “next-step matrix” [21] was constructed. This is illustrated in the right part of Fig. 2. Also, a validation of the analysis and conclusions on the basis of the matrices [21] were performed through repeated discussions with a researcher (AL) having extensive qualitative expertise and who had not been involved in the development of the interview guide or the conducting of the interviews. Any disagreement in interpretation of data was discussed until a consensus was reached. Lastly, all authors of the article discussed the results of the analysis.

In the presentation of the results quotes were translated from Danish to English by the authors. A cut-off was used so that least two statements from different organisations were needed to provide support for a given opinion/reflection before reported, and when more than half of the answers support a given opinion, the term majority is used. During some interviews, a number of questions were left out due to lack of relevance or low priority given the variation in available time for the interview. Thus, this article was based on questions that were answered in most interviews while a few questions with few reflections were not included in the analysis.

## Results

In the following, the results of the analysis are presented according to the four previously outlined themes. However, due to overlap between themes, some questions are included under a different theme than in the interview guide (Table 1). Explicit excerpts from the interview notes supporting the results below are presented in [Additional file 1]. Standard project management terminology and tools are assumed known to readers and definitions and examples of templates for management tools might be found in any project management literature, e.g. in Olsson and Ahrengot [27].

### Theme 1: Context

A total of 25 individuals representing eleven Danish organisations participated in the interviews. All invited organisations chose to participate; three organisations (H1,H2,H3) representing the public hospitals; two somatic hospitals and one psychiatric hospital. Eight organisations represented the private sector: five organisations from the pharmaceutical industry (P1,P3,P4,P5,P6) and three (P2,P7,P8) from the medical device industry.

In the private sector, most respondents were from senior management and interviews were almost exclusively performed with only one individual. In the public healthcare sector, interviews included on average four individuals; the majority being junior staff with limited experience. The number of R&D projects (IMTs) in the pipeline varied greatly at the time of interview and based on answers from eight organisations the range was 20 to 350. Based on six organisations the time horizon for developing a product ready for launch in the market or adoption for routine use ranged from 10 years to develop a product down to 2 weeks to develop an app.

### Theme 2: Foundation or basis for decision-making

Various criteria or domains are used to make stop/go decisions and organisations state that they use a mix of quantitative and qualitative methods for assessment. The following five main criteria are stressed:

- Strategic fit/misfit vis-à-vis the strategic aims of the organisation (H1,P3,P4,P7)
- Clinical effects or technological aspects, including target product profile (P3,H3,P4,P5,P6,P7) or competition and pro/cons of competing technologies (P2,P3,H3,P6,P7,P8)
- Patient or customer perspective (H1,P2,H3)
- Organisational issues including staff, education (H1,H3,P7)
- Business cases or cost analysis (H1,H2,H3,P1,P2,P3, P4,P5,P6,P7,P8), market analysis or size of target group (H1, H3,P1,P3, P4,P5,P6,P7), needs assessment (P5,P7,P8), patents (P3,P5)

All organisations indicate the use of risk or uncertainty assessment, and traditional project management tools for risk assessment, including probability of an event multiplied by impact (H2,P1,P3,P7), were popular. However, the degree to which the organisations believe risk/uncertainty assessment to be important seems to vary from low in the public sector (H1,H2) to very high in most private organisations (P1,P3,P6,P7). Example of other tools used in risk/uncertainty assessment and attitudes towards risk assessment are presented in [Additional file 1].

### Theme 3: Process and structure of early assessment

Most organisations are very structured in their assessment process. The majority indicate having an approach or template for doing early assessments (H1,P1,P2,P3,P4,P5,P6,P7,P8) - two organisations have not (H2,H3). When asked about how systematic their early assessment approach is, answers range from very systematic (P1,P3,P5,P6,P7,P8) to not so systematic (H1,H2,P2,H3,P4).

How the interviewed organisations decide which attributes or effects to include range from very ad-hoc (H1,H2,P2) to more structured and systematic approaches (P1,P3,P4,P5,P6,P7). Some mention having a tool or template as a point of departure (H1,P5,P7). P2 note that they “assess from a case-to-case basis”. An example of a more structured approach is P3, who note that “much is decided by authorities, guidelines”.

#### Phases

The use of a phase model in the developmental process is prevalent and often standard models are used, e.g. tools from project management (tracking milestones, PRINCE2 model), proof of concept models, stage-gate models, or the phase 1–4 terminology used in clinical research when developing pharmaceuticals. Two public organisations (H2,H3) indicated no use of phases in the developmental process. The remaining organisations all use a phase model ranging from very detailed models (H1,P1,P3) to more simple (few phases) or they use standard project management or innovation models like the “Technology Readiness Levels” model, cf. Figure 1 (P2,P4,P5,P6,P7,P8).

#### Updating and learning from past experiences

The majority update their assessments regularly over the development phases, while it varies how often and how stringently, but the idea of continuously learning is prevalent. Two public hospitals (H2,H3) do not mention updating their assessments regularly – the rest say that they do. Five private organisations (P3,P5,P6,P7,P8) explicitly mention time intervals for updates ranging from monthly, to every 6 months, to once a year. Some private organisations actively use past information to qualify the assessments of new technologies (P3,P4,P6). Examples are historical probabilities of success in assessment of new IMTs, cost estimates, probability for phase transition, etc.

#### The use of a formal go/no-go committee

The majority of organisations (H1,P1,P2,P3,P4,P5,P6,P7,P8) have formalised stop/go decisions in the process of development, e.g. stop/go is made (almost) entirely in a formal decision committee or board dedicated to priority setting and making go/no go decisions. However, two public hospitals (H2,H3) have no formalised stop/go decisions in the process of development but have what they call “organisational” stop/go. Note on H2: *“No – but organisational stop/go, i.e. if there is no ownership in a department, development can be brought to an end. There are no systematics, however. If a department has access to financing, they can just continue the process”*. The innovation unit or project staff to some degree decide go/no-go in the three public hospitals (H1,H2,H3) – and if they do not decide “organisational” stop/go comes into play. In contrast to this organisation P1 has detailed descriptions of all meeting activities in relation to the product development, e.g. aim, standard agenda, frequency, participants, and their roles and responsibilities in/before the meeting, etc. In the private sector, stop/go decisions are made almost entirely in formal committees or boards dedicated to prioritising and often consisting of senior management or Chief Executive Officer level (depending on the magnitude of the decision). Examples are: Portfolio board, product review committee, pipeline-forum, and product priority group.

#### Success rate in product development

**Fig. 3:**
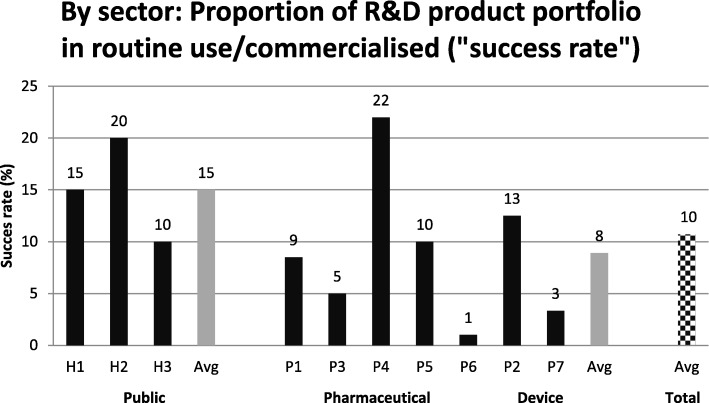
Success rate: Proportion of product portfolio being commercialised/launched or achieving routine use, *N* = 10. Note: Each black column represents the answer from one organisation. The grey columns are calculated averages in the public and private sector. The checkered column is the average for all ten organisations

Figure 3 displays the self-reported success rates (definition provided in method section) of ten organisations. On average, only 10% of the IMTs end up being a success. In the public sector, the figure is 15%, while the private sector on average reports a success rate of 8%. The success rates ranged from 1 to 22% indicating considerable variation, especially intra-sector variation in the private sector ranging from very low rates around 1–5% to as high as 22%.

### Theme 4: Perceptions

The relevance and satisfaction with the applied early assessment practice range from low (or not having an approach) in two out of the three public hospitals (H1,H2) to those stating some room for improvement (H3,P2,P3,P6) over very useful or satisfied (P1,P4,P7,P8). Further, no pros are mentioned by the public hospitals while the private highlights three pros of their early assessment practice: 1) control and standardisation (P6,P7), 2) transparency and ease of communication (P3,P4,P7) and 3) critical questioning and challenge of assumptions (P1,P5,P7).

Critical questioning and control organs regarding inputs and the process surrounding early assessments are pervasive in the private organisations (P1,P2,P3,P4,P5,P6,P7). Further, there is a demand for challenge of assumptions in about half of all the studied organisations while mainly the public sector would like to have more structure and clear goals. Four cons (and suggested solutions in some cases) are mentioned by the studied organisations. First con is more structure and clear goals/KPIs (key performance indicators) (H1,H2,P2,H3). Second con is a lack of challenge of assumptions, i.e. internal and external scrutiny is necessary regarding inputs in assessments. Specifically external challenge/feedback by people without vested interests (H3,P2,P3,P5,P7) or broader stakeholder involvement in the form of early advisory team or workshops (H1,P8). Third con is better needs assessment and pre-qualification/early selection, e.g. achieved by risk sharing (H1,H3,P3,P8). Fourth con is a need for experienced people and strategic decisions like stop/go for IMT development made on the right organisational level by the right people, i.e. not decided by development or project staff (H3, P6).

### Theme 5: Handling cognitive biases

This theme emerged during data analysis. A range of statements seemed to revolve around the concept of cognitive biases. Two cognitive biases, optimism bias and cognitive load, and two approaches to minimise them receive particular focus, predominately in the private sector.

There is great understanding for the importance of handling optimism bias (H3,P1,P2,P5,P7). Optimism bias is sometimes countered by using historical data on probability of success, by using a devil’s advocate process to test assumptions, by using pessimistic assumptions, etc. The second cognitive issue may be called cognitive load. Cognitive load refers to any demands on working memory storage and processing of information [28]. When the load exceeds the capacity of the person processing it causes cognitive overload. Interviews show that gut-feelings/simple heuristics and experience play an important role in early assessments in the private sector (P1,P3,P5,P6,P7).

## Discussion

This study with interviews of 25 participants from a total of 11 organisations investigated how early assessment is performed in different health organisations to draw learning points for the development of an early assessment model for hospitals. The actual development and presentation of an early assessment model for hospitals is presented elsewhere [25]. Analysis identified four areas where early assessment is similarly performed across the studied organisations and four areas where differences exists in how early assessment is performed or perceived between public and private organisations. The four general features across organisations regarding how early assessment is performed are:

1. Traditional project management tools like probability of an event multiplied by impact is popular to assess risk or uncertainty.
2. A broad set of criteria are used and both quantitative and qualitative methods are used to assess them.
3. Critical questioning and challenge of assumptions are central (and often wanted when not present), i.e. internal and external scrutiny is necessary regarding inputs in assessments, specifically external feedback by people without vested interests.
4. Perceived cons are noted and include better assessment of needs, more experienced individuals, and strategic decisions including stop/go for IMT development should not be decided by development/project staff.

In four areas differences exist in how early assessment is performed and perceived in private organisations and public hospitals. In contrast to the situation in the private organisations the following results were found in the public hospitals:

5. Less or no use of: a phase model, stop/go committees, and updates, in the developmental process.
6. Less focus on risk assessment and handling cognitive biases.
7. Lack of structure and clear goals/KPIs, few indicate having an approach or template for doing early assessments, and which attributes or effects to include are ad-hoc.
8. Less satisfaction with current early assessments practise. Further, no pros are mentioned which is in contrast to private organisations highlighting three pros of their early assessment practice: 1) control and standardisation, 2) transparency and ease of communication, and 3) critical questioning and challenge of assumptions.

The above findings carry implications for early assessment in hospitals. Given that hospitals list no pros and are less satisfied with current early assessments practise than private organisations lead us to believe that some of the above findings present relevant learning points for hospitals. Based in particular on bullet 7 and 8, it seems that a more formal and critical early assessment model is needed in hospitals rather than an ad-hoc practise observed in this study. Thus, more in accordance with private approaches two issues seem particular promising when organising and performing early assessment in hospitals. Firstly, having dedicated prioritising committees for IMTs making stop/go decisions. This committee is separate from the IMT development processes and involved staff. The committee might meet regularly, and consist of senior management and external/internal individuals without vested interest in the IMTs. The committee should nurture a culture of critical questioning of assumptions behind the early assessments (bullet 3, 4 and 5). Secondly, supporting the committee members in their stop/go decisions an early decision support tool is suggested with the following five characteristics: 1) transparency, 2) a broad set of domains using both quantitative and qualitative measures, 3) iterative, i.e. continues measurements with a limited number of KPIs, 4) describes uncertainty, and 5) minimise cognitive biases. The above characteristics are based in particular on the findings in bullet 1, 2, 5, 6, 7 and 8.

### Comparing results with the literature

The usefulness of iterations and frequent updates (on KPIs) found in our study is supported by Besner and Hobbs [29]. They find differences in level of use in innovative and non-innovative projects regarding: updated business case at gates and monitoring critical success factors. Further, in the project management literature it is common that the development of products is controlled with a phase or stage-gate model and milestones [27], which our study confirms.

This study found some use of historical data in private organisations, including electronic knowledge databases. Further, stakeholder analysis and risk analysis are often applied. These findings are supported by Besner and Hobbs [29] identifying significant differences in the way innovative and non-innovative projects are managed. Based on answers from 734 mainly program directors or project managers, they find that tools with the greatest differences in level of use between high and low performers include: 1) database for cost estimation, 2) stakeholder analysis, 3) database of historical data, lessons learned and risks, 4) value analysis, 5) medium-term post evaluation of success, and 6) project mission statement. From this study it seems that public hospitals exhibit some of the traits of low performers, e.g. a lack of focus on updates and risk assessment, unclear goals, and they rarely use lessons learned from other projects (databases of historical data). Hence, reviewing project performance may not work well in the public hospitals challenging the ability to perform gap analysis, which is an important warning sign in identifying low potential IMTs [30].

Our study demonstrates the importance of critical questioning and internal and external challenge of used assumptions in the early assessment. Ballini et al. [31] used a multidisciplinary panel inviting “sceptics” to contribute to the early assessment process. Bonabeau, Bodick and Armstrong [32] recommends a segmented approach to new product development with two phases: “an early stage that focuses on evaluating prospects and eliminating bad bets, and a late stage that maximises the remaining candidates’ market potential”. In this approach, the early-stage organisation maintains loyalty to the experiment rather than to the product. Hence, both studies underline that you cannot objectively review your own product but need assistance.

In our study, the reported average success rate is 10% across organisations (15% in the public vs. 8% in the private). However, stating a success rate is not trivial and depends on many factors. One factor influencing the success rate is what kind of technologies the organisations have chosen to include when answering the questions. It is likely that the IMTs in the public sector are more mature on average compared to the private IMTs and as a consequence they have a higher success rate. Other differences in IMT characteristics include the development period for the included IMTs, which varies a lot. In the literature, success rates, i.e. the probability of commercial success rate, for early stage private ventures are reported to be on average 11% [33]. In pharmaceuticals, where the lead time can easily reach 15 years, there is an overall success rate of only 0.002% and the company tracks the success rate and duration in every phase from discovery to marketing approval [30].

This study identified two cognitive biases and the importance of actively handling these. Cook [34] handles the topic of overconfidence in judgmental forecasting and lists six principles that forecasters should heed in order to counteract overconfidence. Our findings are consistent with several of these principles underlining the importance of tackling overconfidence, i.e. optimism bias, in early assessment.

### Strengths and limitations of this study

This study is to our knowledge the first to explore how early assessment is carried out in different health organisations, with the aim of identifying leaning points for hospitals. The study constituted the basis for and partly guided data extraction in a literature review of early assessment [22] and might inform further studies in the field. As the study is exploratory and restricted to a Danish context the ability to generalise the findings is somewhat limited. Other limitations include a decision for interviews to be confidential and not tape recorded which was deemed necessary to gain access to key persons in a competitive and secretive area. However, this choice was based on literature and without in practice investigating whether resistance was present towards recording and transcribing the interviews. In retrospect this ought to have been explored explicitly.

Participants were deliberately chosen to reflect a high level of variation adhering to the principle of maximum variation where the researcher selects a small number of cases that maximize the diversity relevant to the research question [35]. The interviewed organisations matched the goal of large variation, both regarding number of products in pipeline, time horizon in product development, success rate, and experience with early assessments including position of participants in the organisations.

Large organisations in the pharmaceutical industry, medical device industry, and public hospitals were preferred for inclusion since more sophisticated or cutting-edge methods might be more likely found in larger organisations with distinct departments for research portfolio analysis and planning. The included private sector organisations were chosen because of extensive experience regarding R&D investments. An annual Industrial R&D Investment Scoreboard published by the European Commission [36] shows that the pharmaceuticals & biotechnology sector is leading in R&D investments and therefore may be assumed to be skilled to make early assessments regarding R&D investments. However, the possibility exists that including for example small companies, experience from venture capitalists, or “incubator helpers”, could have provided valuable input. Venture capitalists are considered experts in identifying high-potential IMTs and their ventures survive at a much higher rate than ventures backed by other sources [37].

### Transferability of results and future research

Transferring results from private sector approaches to a hospital context requires adaptations to be relevant for the hospitals’ decision problem. First, the criterion for decision making may be different, e.g. commercial potential and profitability vs. clinical potential, benefit to patients, and costs. Also, the time horizon in product development differs in the included sectors. Further, in the private sector, IMT development (innovation) is the cornerstone of the business in contrast to the public hospitals where this activity naturally is secondary to the main task of delivering established healthcare services. Given the above differences, the more formal (or professional) practice observed in the private sector regarding early assessment is not surprising. However, since resources spent on innovation (IMTs and projects) are rising in the public sector, we believe that the private sector approaches is increasingly relevant for a public context. Further, the included sectors are familiar with clinical effects and often also the cost-effectiveness concept, which is considered positive for transferability of results to a hospital context.

The context theme seemed to indicate a possible difference in experience level between hospitals and private companies. Thus, a direct question on number of years employed with early assessing IMTs would have been relevant to include. With such information an experience ordered matrix could have been constructed which may have yielded interesting analysis results. Moreover, it may be interesting to explore the higher self-reported success rates in public hospitals compared to the private organisations. The concept of a success rate was well known and used in private organisations and they often had a straight answer and a practice of tracking this measure. Public hospitals needed more explanation and the concept seemed less familiar and used. We believe it relevant with future research into whether possible differences exist in how the success rate is perceived and used in the two sectors. Also, more efforts could have been directed on how the different organisations might have different requirement and concerns at different stages of technology development from early stages and onwards. Finally, an open question on how early assessment is defined in each of the interviewed organisation would have been relevant.

## Conclusions

In this study similarities and differences in the way early assessment is done in different health organisations was identified. Based on this, two learning points in particular seemed relevant for hospitals. Firstly, having dedicated prioritising committees for IMTs making stop/go decisions. This committee is separate from the IMT development processes and involved staff. Secondly, the committee should base decisions on a decision-support tool with five characteristics (transparency, a broad set of domains, iterative, describes uncertainty, and minimise cognitive biases). Thus, findings from this explorative study are believed to carry promising elements for the development of an early assessment model adapted to hospitals.

## Additional file

Additional file 1: A conceptual framework for design of the interview study and statements from the response data supporting the results of each theme are presented. (DOCX 32 kb)

## Abbreviations

HTA: Health Technology Assessment
IMT(s): Innovative Medical Technologies
KPI: Key Performance indicator
R&D: Research and Development
