# Supplementary material for: A qualitative exploration of early assessment of innovative medical technologies
Source: BMC Health Serv Res. 2018 Nov 6;18:837. doi: 10.1186/s12913-018-3647-z (PMC6220450; doi:10.1186/s12913-018-3647-z)
Supplement: Supplementary file 1 — A conceptual framework for design of the interview study and statements from the response data supporting the results of each theme are presented. (DOCX 32 kb) [file 12913_2018_3647_MOESM1_ESM.docx]

# Additional file 1

## A conceptual framework

Overall, we wish to identify how early assessment is performed and perceived and whether any differences/similarities are present between the included sectors. Further, we wish to discuss if there are areas where it seems relevant that hospitals gain inspiration from private approaches, i.e. are any learning points for hospitals identified? Thus, a conceptual framework was developed to cover a study on the above topic, see Figure 4.

Figure 4 Conceptual framework for a study on early assessment

|  | **Context** (sector – private/public, type of IMT, number of IMTs in portfolio, time to develop IMT) | |  |
| --- | --- | --- | --- |
|  | **Process**   - How decentralised is decision making regarding further development? (is there stop/go – and who decide stop/go to development) - How “tight” or focused is the development process? (phases, updates, success rate) | **Perceptions**   - Pro/cons - Satisfaction |  |
|  | **Foundations**   - Criteria / domains - Methods used to value domains |  |  |
|  |  | |  |

## Statements from the interviews

Statements from the response data supporting the results regarding theme 2 to 5 are presented below.

**Box 1. Examples of statements relating to risk** **or uncertainty assessment (theme 2)**

| **Element** | **Examples of statements presented** |
| --- | --- |
| Private | P1: Do a pipeline valuation, i.e. an overview of the total value of their portfolio of products where they implicitly take risk into account. As the completion of a specific product goes up, so does its value in the portfolio. In other words, they use progress through the phases when controlling and evaluating their products.  P3: They have a sector/department dedicated to performing risk assessment.  P3: “What-if”-scenario analyses to test sensitivity and sometimes SDO (strategic development options) if they have to make bigger strategic decisions.  P6: Focus is on minimising risk all the time.  P6: Sometimes this was visualised with a “traffic light” approach using the colours to indicate how serious a given issue is perceived to be.  P7: “Risk burn down” when starting a new project decomposing risk into economic, organisational, and technical risks and based on these inputs they do the following: Perform an aggregated risk evaluation (defined as a percentage) for the project in total. A burn-down process is applied over time. Include a line tracking for expected and actual decrease in uncertainty.  P7: They specify the risk at each tollgate in a PowerPoint presentation. This is a set of standard slides that all projects must fill out. What are these slides based on, who did you ask? This information confers a solid platform for deliberations. Mostly a qualitative description. |
| Public | H1: It [uncertainty] is qualitatively described. They do not see themselves as risk managers.  H2: In the project description – but that is a recent phenomenon |

**Box 2. Examples of statements relating to the process and structure of early assessment (theme 3)**

| **Element** | **Examples of statements presented** |
| --- | --- |
| Updates | P2: Yes, iteratively.  P6: Yes, every six months. Update the assessments every time they move on.  P5: Yes, all the time – but on a lesser scale. Annual assessment in the R&D board – more frequently at a below-board level. |
| Learning from past experiences / use historical data | P1: Yes – open the case after three years. Make the same mistakes again if they don’t do this. They have learnt from the quality staff, GMP (good manufacturing practice).  P3: Use benchmarking: They use probabilities from a shared anonymous benchmark database (international database with only big-pharma – 10-20 companies) |
| Use of a formal go/no-go committee - private | P4: Portfolio board with people from global and local departments (they prioritise, clean out). Country manager + head of regulatory affairs/expenses are part of the board. The country manager is not involved in all products – some decisions are considered minor. High transparency in the organisation regarding who decides.  P5: Their department collects and distils information for the R&D board – having a structure for the decision-making is important.  P8: Product committee with the entire manager group (owner, head of finance, quality, product, purchasing, etc.). Decide together, but in the end the owner has the final say. Always the same individuals in this central group. Meetings monthly. Projects are approved at every defined milestone. |
| Use of a formal go/no-go committee - public | H2: No – but “organisational” stop/go, i.e. if there is no ownership in a department, development can be brought to an end. There are no systematics, however. If a department has access to financing, they can just continue the process.  H3: As long as financing was available, the decision was made by [name of interviewed consultant], who decided what continued – it should have been the management. |

**Box 3 Examples of statements describing pros of their early assessment process (theme 4)**

| **Types of pros** | **Examples of arguments presented** |
| --- | --- |
| 1) Control and standardisation | P7: Highly creative mind setters are challenged by the strong structure (“accounting” approach it was called repeatedly). Must love, and endorse, working in a systematic and uniform manner. |
| 2) Transparency and ease of communication | P4: There is a high level of internal transparency in relation to data access and processes. In the launch department, a number of Access databases with all the different information/data are available. Staff from regulatory, lawyers, economists, pharmaceutical staff, etc. are all able to insert information so that everyone has access to all information.  P3: Despite the complexity, it is communicable. Assumptions should be clear and visible. [name of department] provides a nuanced picture, they constitute the “devil’s advocate” for the projects. However, they do so in a transparent way – not by applying complex calculation methods. |
| 3) Critical questioning and challenge of assumptions | P5: For this reason, the system/setup includes different decision levels, iterative scrutiny. This is to facilitate the internal competition, thereby improving the rate of success for our projects.  P1: Much more selective these days – by the use of these tools. |

**Box 4 Examples of** **statements describing cons of their early assessment process (theme 4)**

| **Types of cons** | **Examples of arguments presented** |
| --- | --- |
| 1) Structure and clear goals/KPIs (key performance indicators) | H1: It is often a bit “messy” with the goals…they are rarely SMART. And after (when the idea leaves H1) there is no clear process to continue measurements.  H2: They often need to articulate or define the success criteria after the project is started.  P2: Start doing more writing down. More concrete on ambitions making it easier to get a common understanding.  H3: Others will have to do the systematic stuff – those with the innovation gene are not able to provide structure. All projects must be assessed on the same parameters including a shared overview of all projects.  P7: Difficult to choose the correct variables (KPIs). But make sure that you have only a few. When working with a portfolio, they usually have only 10-20 KPIs. |
| 2) Challenge of assumptions | P2: Proving people wrong – test assumptions + designers shouldn’t be part of the evaluation themselves - they are too vested. This happens often.  P3: Use “internal challenge”, e.g. on used assumptions.  P5: Someone within the organisation verifies input – a culture of scepticism. It is central with an unbiased view on things. Those who rise in the pharmaceutical industry know that it is vital to kill ideas…. You need to falsify. That is a normal way of thinking.  P7: Plans are reviewed based on: technical review, plan-review (other project managers), clinical review (scientists), advisory boards (external people, i.e. advisory boards are used on all products!). They ask questions: Why should we not do this – both technical and economic questions.  P2: He is very positive about their new collaboration with venture people – there is a need to be critical right from the beginning.  H3: It is dangerous to evaluate yourself, e.g. use spring-boards to assess new products. They ask critical questions. It costs money to do so, but they are probably well spent.  P5: You can’t trust the project – it just wants to survive!  P7: Push-back is important - you are seduced by your own thoughts. |
| 3) Better needs assessment and pre-qualification/early selection + the idea of risk-sharing | H1: They go with more than they should. Better pre-qualification. Substitute the subjective with the objective is what [name of boss] calls it. They are missing a clear “value-proposition” (having the need affirmed), e.g. the hospital wants to invest x money in this.  P3: They use royalties and milestone payments when they cooperate with other partners, i.e. they link payment to success.  H3: Choose from strategic reasons. Innovation must be driven by needs. Hence, the clinical departments must be willing to pay or the project should be “killed”. Ask the clinical department: do you want to pay the running costs of say 29,000 monthly? If not, you should not develop this app! 9 out of 10 will not do so. |
| 4) Inexperienced people + strategic decisions made by development people | H3: Recruit inexperienced people to the area of innovation – it is a long education. The same is the case in management (they don’t have the competencies, do not know enough). Random promotions/appointments.  H3: “Project-makers” put things in motion – without always thinking about the strategic/clinical need.  P6: Historically, development has been strong (too strong!). The “no-people” (commercial people) have not been strong enough. |

**Box 5. Examples of statements describing** **cognitive biases in the early assessment process (theme 5)**

| **Type of bias** | **Examples of arguments presented** |
| --- | --- |
| 1) Optimism bias | P1:”Dreamers” have a short lifespan here. They are very expensive. + They only model, extrapolate. They are conservative. Only a minimum of guessing/guesswork involved.  P1: A flaw within the internal project overview. There is a wish to see the project survive, or to “hype” it. But this was not possible, and not something that should be done!  P5: You can’t trust the project – it just wants to survive!  P7: Push-back is important - you are seduced by your own thoughts. |
| 2) Cognitive overload including how gut-feelings, experience and simple heuristics play an important role in minimising the load | P1: They are much more quick and dirty these days – an extremely structured gut-feeling.  P3: It is vital that the entire organisation understands the evaluation. It cannot be a black box. For example, individuals from the finance section doing ”real options”, etc. [Name of department] are open about their assumptions in the evaluations.  We guess – but it is based on this. It is better to do simple calculations and be able to explain why. That creates transparency + we will not signal more precision than what is real.  P6: Rather vaguely right than precisely wrong.  P6: “Black box” if the assessments are too advanced (mathematically) – so they don’t use that. They must be able to communicate it to the entire organisation. Sometimes an economist cannot even remember how their model, e.g. an advanced CEA, is constructed.  P3: [name of department] is a senior group (very experienced individuals). You need to understand most of the “value-chain” to make these assessments.  P6: Important with experienced people in the assessments. No mathematics – it makes no sense. It all comes down to gut-feeling or experience. |
